# The Involvement of Heat Shock Proteins in the Establishment of *Tomato Yellow Leaf Curl Virus* Infection

**DOI:** 10.3389/fpls.2017.00355

**Published:** 2017-03-16

**Authors:** Rena Gorovits, Henryk Czosnek

**Affiliations:** Institute of Plant Sciences and Genetics in Agriculture, Robert H. Smith Faculty of Agriculture, Food and Environment, The Hebrew University of JerusalemRehovot, Israel

**Keywords:** begomovirus, heat shock proteins, protein quality control, tomato, whitefly

## Abstract

*Tomato yellow leaf curl virus* (TYLCV), a begomovirus, induces protein aggregation in infected tomatoes and in its whitefly vector *Bemisia tabaci*. The interactions between TYLCV and HSP70 and HSP90 in plants and vectors are necessity for virus infection to proceed. In infected host cells, HSP70 and HSP90 are redistributed from a soluble to an aggregated state. These aggregates contain, together with viral DNA/proteins and virions, HSPs and components of the protein quality control system such as ubiquitin, 26S proteasome subunits, and the autophagy protein ATG8. TYLCV CP can form complexes with HSPs in tomato and whitefly. Nonetheless, HSP70 and HSP90 play different roles in the viral cell cycle in the plant host. In the infected host cell, HSP70, but not HSP90, participates in the translocation of CP from the cytoplasm into the nucleus. Viral amounts decrease when HSP70 is inhibited, but increase when HSP90 is downregulated. In the whitefly vector, HSP70 impairs the circulative transmission of TYLCV; its inhibition increases transmission. Hence, the efficiency of virus acquisition by whiteflies depends on the functionality of both plant chaperones and their cross-talk with other protein mechanisms controlling virus-induced aggregation.

## Introduction

Plants often grow in unfavorable environments such as poor soils, heat and drought, and have to cope with pathogens such as viruses, fungi, bacteria, and with sucking and chewing insects. Plants have adapted to these conditions and their genome contain genes conferring tolerance to various stresses, which are tapped by breeders to develop varieties adjusted to these environments. Plants have also developed strategies to cope with diseases transmitted by pathogens. They use either pre-formed structures and chemicals to stop spread and repel invaders or they respond to infection by inducing an immune-like response. Both type of responses sense and react to the pathogen by sending signals to other cells of the plant, leading to transcriptional reprogramming, and biosynthesis of compounds that limits pathogen spread (Jones and Dangl, 2006). Immunity comes in different forms, from PAMP/MAMP-triggered immunity to *R*-gene mediated immunity; often these processes are happening simultaneously. The HR does not always occur in *R* gene responses for pathogens or insects. However, the signaling cascades and downstream gene expression does occur in all interactions in host and non-host organisms. HR acts largely inside the cell by using proteins encoded by *R* genes that cause an apoptotic hypersensitive response, or/and by activating resistance and defense genes. Plant may also respond to infection in one part of the plant enhancing the defense response in other parts (Hail and Bostock, 2002; Fu and Dong, 2013). Against viruses, plants frequently mobilize RNAi-mediated gene silencing mechanisms to suppress the expression of viral genes (Mandadi and Scholthof, 2013).

*Tomato yellow leaf curl virus* (TYLCV) is a begomovirus (genus *Begomovirus*, family *Geminiviridae*) transmitted by the whitefly *Bemisia tabaci* (*Hemiptera: Aleyrodidae*). It is one of the viruses causing the most damages to tomato crops (Czosnek, 2007; Scholthof et al., 2011). The TYLCV complex comprises many species and isolates discernable by their DNA sequence (Czosnek, 2008). Begomoviruses belong to the Geminiviridae family characterized by a 22 nm × 38 nm geminate virion containing one circular ssDNA genome of 2,700–2,800 nucleotides in length. The encapsidated TYLCV genome strand comprises two genes, V1 and V2; the complementary-sense strand (synthesized during the viral DNA replication) comprises four genes, C1 to C4 (Navot et al., 1991). V1 encodes the coat protein (CP), which is indispensable for cell-to-cell and long-distance movement, and transmission by whiteflies. All the other genes are also multifunctional and their activity is aimed at ensuring virus replication and spread, and at counteracting plant defenses (summarized by Díaz-Pendón et al., 2010).

The path of TYLCV (and begomoviruses in general) in the infected plant and in the whitefly vector is known in its broad features. Virions are inoculated into the phloem by viruliferous whiteflies during feeding and transported to the phloem-associated cells. The viral single-stranded DNA (ssDNA) genome is then freed from the capsid. Replication is initiated when host DNA polymerases synthesize the virus genome complementary strand, creating a double-stranded DNA (dsDNA) form of the viral genome. The proteins encoded by the complementary strand are expressed, especially the replication-associated protein (Rep encoded by the C1 gene), initiating the rolling-circle replication mechanism. The CP is expressed and the nascent viral genomic ssDNA is packaged into virions. The viral particles propagate cell-to-cell and long-distance via the phloem (see details in Hanley-Bowdoin et al., 2013). TYLCV-infected susceptible tomato plants are stunted, leaves are curled and swelled, and yields are reduced.

*Bemisia tabaci* acquires TYLCV with their stylets while feeding on infected plants. Then virions reach the esophagus and the midgut, which they cross into the haemolymph on their way to the salivary glands. Secretory cells mediate the transmission of begomoviral particles to plants together with saliva (Ghanim et al., 2001; Wei et al., 2014). TYLCV can express viral genes and replicate in the insect vector (Pakkianathan et al., 2015; Wang et al., 2016).

*Tomato yellow leaf curl virus* is associated with modifications of the expression patterns of many genes, as well as changes in the protein and metabolite contents of both host plant and insect vector. All these changes are thought to facilitate host invasion, virus genome replication and expression, and to resist host defenses. In this article, we summarize our knowledge on the association of TYLCV with tomato host and virus vector chaperone systems, a critical step that ensures a successful infection.

## TYLCV Interactions with Plant Host and Insect Vector Heat Shock and Quality Control Proteins

### TYLCV Infection Leads to Changes in the Transcriptome, Proteome, and Metabolome of the Tomato Host Plant and of the Whitefly Vector

Transcriptome analyzes of tomato infection (using subtraction cDNA libraries and microarrays) revealed that TYLCV induces significant changes in the 1st days after inoculation, changes that exacerbate as infection progresses (Eybishtz et al., 2009; Chen et al., 2013; Sade et al., 2013; Miozzi et al., 2014). These responses include the activation of genes involved in general stress-response, hormone biosynthesis, signal transduction, RNA regulation and processing, induction of the ubiquitination pathway and initiation of autophagy. TYLCV-susceptible plants emitted high levels of reactive oxygen species (ROS), pathogenesis-related (PR), and wound-induced proteins. Sources of carbon and nitrogen were highly affected (Moshe et al., 2012). Tomato infection with TYLCV was accompanied with significant changes in the abundance of various classes of metabolites such as amino acids and polyamines, phenolic and indolic metabolites, indicating a tightly coordinated reprogramming of phenylpropanoid, tryptophan/nicotinate, urea/polyamine, and salicylic acid biosynthesis pathways leading to the production of defense compounds (Moshe et al., 2012; Sade et al., 2015).

Tomato infestation with non-viruliferous whiteflies induced a decrease in the amounts of MAPKs, heat shock proteins (HSPs), as well as increased activities of the *PR* genes, β-1,3-glucanase, and peroxidase. These effects were exacerbated when the insects carried TYLCV (Gorovits and Czosnek, 2007; Gorovits et al., 2007). In another study, it was shown that PR genes are expressed when *B. tabaci* and the greenhouse whitefly *Trialeurodes vaporariorum* are feeding on tomato plants (Puthoff et al., 2010). Transcriptome analyses of different plants (e.g., *Arabidopsis*, Kempema et al., 2007; tomato, Musser et al., 2014; cotton, Li et al., 2016) upon infestation by non-viruliferous whiteflies showed a specific expression of genes associated with photosynthesis, senescence, secondary metabolism, and stress.

The interactions of geminiviruses with their insect host also induced changes in signaling and defense pathways. The long-term presence of TYLCV in the whitefly host (sometimes for the remaining lifespan) has deleterious effects on the longevity and fertility of the insect (Rubinstein and Czosnek, 1997; Pan et al., 2013). In the recent few years, high-through put sequencing has allowed studying the transcriptome of different species (previously referred as biotypes) of adult whiteflies from various locations, males and females, and their developmental stages (Leshkowitz et al., 2006; Wang et al., 2011, 2012, 2013; Seal et al., 2012). In addition to whole whiteflies, the transcriptome of several organs involved in begomovirus transmission such as the primary salivary gland (Su et al., 2012) and the gut (Ye et al., 2014) has been analyzed. Genes differentially expressed upon TYLCV (or *Tomato yellow leaf curl China virus*, TYLCCNV) acquisition and retention were identified by several methods, including subtractive hybridization (Li et al., 2011), microarrays (Götz et al., 2012) and transcriptome sequencing (RNA-Seq) (Luan et al., 2011). Results showed that more than 1,500 genes were differentially regulated. Among these were genes involved in the activation of the immune responses and of the autophagy pathway, as well as genes encoding HSPs. Several studies aimed at investigating the response of *B. tabaci* to plant defenses have shown that the insect is able to detoxify induced secondary metabolites (Alon et al., 2012; Elbaz et al., 2012).

### Interactions of TYLCV and Other Viruses-Encoded Proteins with Host Proteins

*Tomato yellow leaf curl virus*, with only six genes (eight genes in begomovirus with bipartite genomes), needs to replicate, spread and counter host defenses (Hanley-Bowdoin et al., 2004, 2013). For instance, since begomoviruses do not encode their own replicase, they use the Rep protein (encoded by the C1 gene) to interact with the host DNA replication and cell cycle machineries. For example, the *Tomato golden mosaic virus* (TGMV) Rep cooperates with a retinoblastoma-like protein to promote the replication of the TGMV DNA (Arguello-Astorga et al., 2004), while the Rep of *Tomato yellow leaf curl Sardinia virus* (TYLCSV) recruits a complex of proliferating cell nuclear antigen (PCNA) and plant DNA polymerase to the viral origin of replication (Castillo et al., 2003). TYLCV V2 is a Suppressor of Gene Silencing (Zrachya et al., 2007), which interacts with the host proteins SGS3 and CYP1 (Glick et al., 2008; Bar-Ziv et al., 2012). TYLCSV C2 interacts with the COP9 subunit of the signalosome (CSN), a complex involved in the regulation of the ubiquitination, preventing tagging the virus for destruction (Lozano-Durân et al., 2011). TYLCV C4 protein interacts with tomato plant defense proteins (Kim et al., 2016).

The ability of viruses to hijack cellular processes stipulates that the infected cell protects the structural and functional complexity of the virus proteins. Many viruses depend on host chaperones/heat stress proteins (HSPs) for folding, protein quality control (PQC) and maintenance of proteostasis (Mayer, 2005; Nagy and Pogany, 2012). HSPs affect virus expression, replication, and assembly and counter the plant responses to infection (Nagy et al., 2011). HSPs are involved in the assembly of the large virus-induced protein aggregates (coined viral factories, VFs), sheltering the virus, promoting their activity and their multiplication (the characteristics of VFs in mammalian cells have been reviewed by Wileman, 2006, 2007; Livingston et al., 2009; Netherton and Wileman, 2011).

HSP70 and HSP90 are the most frequent chaperons utilized by viruses. HSP90 promotes *Bamboo mosaic virus* replication by interacting with the virus replicase (Huang et al., 2012). Similarly, HSP70 and HSP90 form a 480-kDa multicomponent complex with the *Red clover necrotic mosaic virus* replicase and interact with p27, a viral-encoded component of the replicase complex on the endoplasmic reticulum membrane (Mine et al., 2012). The association of HSP70/HSC70 and HSP90 involves interactions with the HSP90 co-chaperone, SGT1 (for Suppressor of G2 allele of skp1) (Noel et al., 2007). In plants (and animals), SGT1 is essential to the function of many NLR (nucleotide-binding leucine-rich repeat receptor) proteins that induce plant defenses (Liu et al., 2004). SGT1 enhances *Potato virus X* multiplication, while *SGT1* silencing led to an increased accumulation of *Plantago asiatic mosaic virus* in *Nicotiana benthamiana* (Komatsu et al., 2010; Ye et al., 2012). Indeed, SGT1 is involved in PQC by associating with the ubiquitin and 26S proteasome protein degradation complexes (Muskett and Parker, 2003) and by interacting with two COP9 signalosome components (Azevedo et al., 2002; Liu et al., 2002).

The HSP70 family is actively participating in the biology of geminiviruses (Gorovits et al., 2013a). During the live cycle of the bipartite begomovirus *Abutilon mosaic geminivirus* (AbMV), the chloroplast cpHSC70-1 proteins binds to the virus movement protein (MP). *In planta*, cpHSC70-1/MP complexes were visualized at the cell periphery and within chloroplasts, suggesting that AbMV utilizes cpHSC70-1 to move intra- and inter-cellularly (Krenz et al., 2010, 2012). Silencing cpHSC70-1 inhibited AbMV movement, but not replication.

Heat shock proteins are also associated with the circulative transmission of begomoviruses in their whitefly vector. Microarray-based analyses of the *B. tabaci* transcriptome in response to the ingestion and retention of the monopartite TYLCV and the bipartite *Squash leaf curl virus* (SLCV) indicated that the insect *Hsp70* transcription is induced upon virus infection. Immuno-capture PCR (IC-PCR) and virus-overlay protein-binding confirmed the interaction of TYLCV and SLCV CP with HSP70 in *B. tabaci* (Götz et al., 2012). In the digestive tract, TYLCV and HSP70 co-localized exclusively in the insect filter chamber and cecae. Whiteflies membrane-fed with anti-HSP70 antibodies had enhanced capacities to transmit TYLCV, indicating that HSP70 limits virus transmission, possibly moderating some of the potential long-term harmful effects of the virus on the whitefly.

Cytosolic HSP70 isoforms were shown to be required at distinct steps of the life cycle of Dengue virus (DENV, genus *Flavivirus*, family *Flaviviridae*), a mosquito-borne virus causing a life-threatening disease in human (Bhatt et al., 2013). DENV generates a web derived from the ER (Welsch et al., 2009), where replication takes place. These processes are highly dependent on the proper folding of viral proteins and its control by cellular chaperones. Moreover, it was shown for the *Japanese encephalitis virus* (a *Flavivirus* related to DENV) that HSP70 protects proteins from degradation (Ye et al., 2013). HSP70 is involved in DENV entry, RNA replication, and virion biogenesis. Nine distinct DNAJ cofactors (also known as HSP40) are necessary for proper HSP70 function: DnaJB11 promote viral RNA synthesis, while DnaJB6 in concert with the CP promotes assembly of viral particles (Taguwa et al., 2015).

### TYLCV Infection Is Characterized by the Induction of Aggregates of Increasing Size, Reminiscent of Animal Viral Factories

It has been known for several decades that Azure-A stains aggregates/inclusion bodies that could be visualized with the light microscope in the phloem-associated cells of leaves of begomovirus-infected (including TYLCV) susceptible plants (Christie et al., 1986). The role of these aggregates in the process of geminivirus propagation and in the host immune response was intriguing.

**FIGURE 1:**
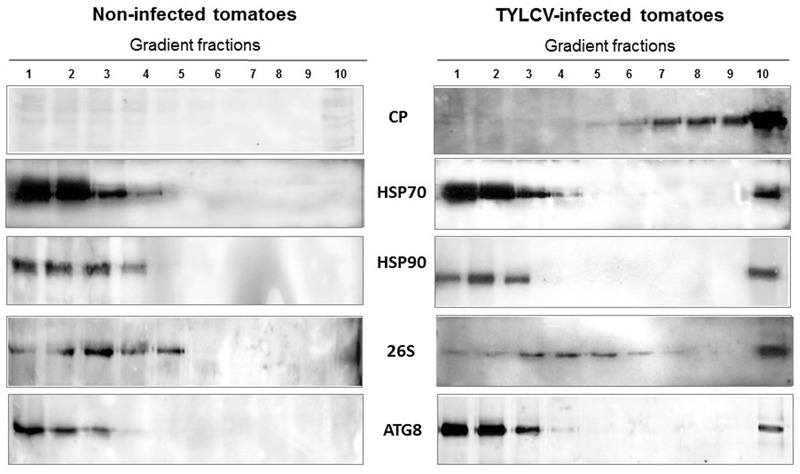
***Tomato yellow leaf curl virus* (TYLCV) induces the aggregation of coat protein (CP) as well as of tomato HSP70 and HSP90 in infected tomato leaves.** Distribution of TYLCV CP and plant HSP70 and HSP90 after sedimentation of leaf native proteins on linear 10–50% sucrose gradients (according to Gorovits et al., 2013b). Leaf homogenates were prepared from infected tomato plants at 28 dpi; non-infected plants of the same age were similarly processed. Gradients were divided into 10 fractions, 1 (top – contained soluble proteins) to 10 (bottom – contains large protein aggregates), and aliquots were subjected to SDS-PAGE, followed by western blot immunodetection with antibodies against TYLCV CP, and plant HSP70 and HSP90.

Ultracentrifugation of native proteins in linear 10–50% sucrose gradients allowed to separate proteins aggregates according to size, from soluble and small (top fractions) to large bottom fractions, via mid-size (Gorovits et al., 2013b). Using *in situ* immuno-detection, cell fractionation and separation of proteins by ultracentrifugation, it was shown that TYLCV CP is localized in aggregates of increasing size as infection progresses (**Figure 1**). These aggregates occur first in the cytoplasm then in the nuclei of phloem-associated cells (Gorovits et al., 2013b). The large CP aggregates, which can be compared with VFs in animal cells (Wileman, 2006, 2007), is a major feature of a successful TYLCV infection. The role of small/mid-size aggregates in sheltering TYLCV components and protecting them from host degradation has been shown in plant and in insect cells (Gorovits et al., 2016). In both hosts, the proteolytic activities in the small/mid-size aggregates were low. At the beginning of plant and vector infection TYLCV proteins were found in mid-size aggregates. Altogether, aggregation may have a dual role: (1) from the virus point of view: protecting the virus from host proteases, and concentrating enzymes and other factors necessary for its replication, (2) from the host plant point of view: sequestrating virus components, isolate and neutralize its proteins, preventing virus expression and prepare viral components for destruction.

### Co-localization of TYLCV CP and Host HSPs

HSP70 presents opposite behaviors in whitefly and in tomato in the presence of TYLCV. Microarray-based analyses showed that the insect *Hsp70* was upregulated as TYLCV is ingested (Götz et al., 2012). In plants, TYLCV does not induce the expression of *Hsp70*. On the contrary, increasing amounts of viral DNA and CP were accompanied by decreasing amounts of plant HSP60, HSP70, and HSP90 (Gorovits and Czosnek, 2007; Gorovits et al., 2007; Moshe et al., 2012).

**FIGURE 2:**
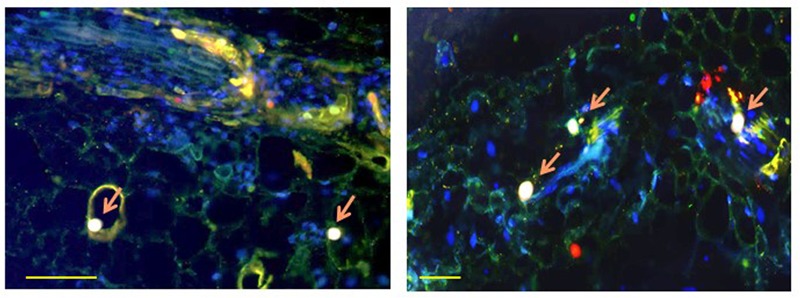
**Co-localization of TYLCV CP with tomato HSP70 (left) and with tomato HSP90 (right) in infected leaf at 49 dpi, as observed with a confocal microscope.** Cross-section through the leaf blade. CP appears as red, cellular HSP70 or HSP90 as green, nuclei as blue; CP co-localizing with HSP70 or HSP90 in nuclei as pink (pink arrow). Bar is 100 nm. The left photograph is reproduced, with permission, from Gorovits et al. (2013a).

**FIGURE 3:**
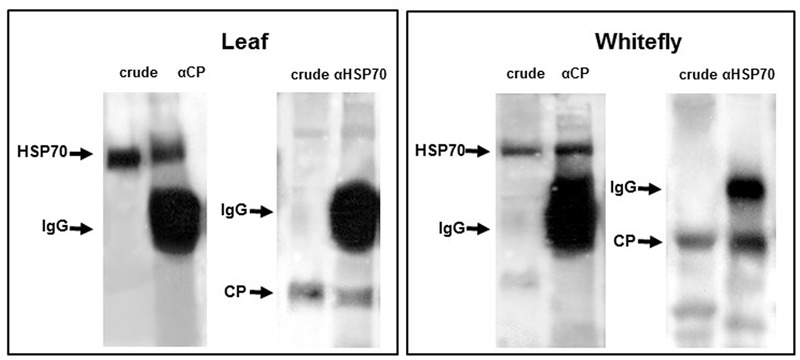
**Co-immunoprecipitations of cellular HSP70 with viral CP and *vice versa* in tomato leaves and *Bemisia tabaci*.** Co-immunoprecipitation of CP with anti-TYLCV CP specific antibody and HSP70 with anti-HSP70 specific antibody in leaf and whitefly protein extracts. The direct immunodetection was designed as “crude.”

HSP70 and viral CP in tomatoes and whiteflies was investigated *in situ* using fluorescently labeled antibodies. In leaves, HSP70 and CP aggregates of increasing size were found first in the cytoplasm then in the nucleus (**Figure 2**). At the late infection stages (49 dpi), the large aggregates contained both proteins (Gorovits et al., 2013a). Co-immunoprecipitation (Co-IP) assay revealed the ability of CP to interact with tomato and *B. tabaci* HSP70 (**Figure 3**), pointing on the development of potential complexes between TYLCV CP and HSP70 of both viral hosts.

Fluorescence microscopy detected co-localized TYLCV CP with HSP90 in leaves of infected tomato, first in cytoplasmic, then in large nuclear aggregates (**Figure 2**). Prior to infection, HSP90 was not found in large aggregates (Moshe et al., 2016). Co-IP showed that plant HSP90 and CP interacted in protein extracts from the nucleus, but not from the cytoplasm. Therefore, HSP70 and HSP90 formed complexes with TYLCV CP in large nuclear aggregates operating as VFs, but not in cytoplasmic aggregates. Interestingly chaperones such as HSP60 and the glucose related protein 78 (GRP78 or BiP) were not found in CP-containing large nuclear aggregates.

In whiteflies, fluorescent *in situ* hybridization and immuno-histology showed that TYLCV CP and HSP70 co-localized in the midgut epithelial cells. IC-PCR, protein Co-IP (**Figure 3**), and virus-overlay protein-binding assays pointed not only on possible co-localization, but also on CP-HSP70 interaction (Götz et al., 2012; Ghanim and Czosnek, 2016). The HSP90 localization and its relation with CP in whiteflies is currently being examined.

### Decrease of HSP70 and HSP90 Affects Differently the Accumulation of TYLCV in Plants and Insects

In plant cells, the expression of *Hsp70* can be reduced by quercetin, a bioflavonoid that inhibits *Hsp70* transcription (Wang et al., 2009). Quercetin-treated infected tomato leaves contained reduced amounts of virus, decreased CP quantities in large nuclear aggregates and increased CP levels in cytoplasmic mid-size aggregates (Gorovits et al., 2013a). Taken as a whole, we propose that HSP70 plays an important role in the nuclear CP transportation and in TYLCV replication.

The involvement of HSP90 in TYLCV infection was studied using the benzoquinone antibiotic geldanamycin (GDA), which inhibits the activity of HSP90. Tomato leaves were treated with GDA and the location of TYLCV CP was examined. In contrast to HSP70, HSP90 did not affect the nuclear localization of CP and, therefore, is not required for the translocation of CP to the nucleus. However, silencing of *Hsp90* and *SGT1* led to enhanced accumulation of TYLCV CP as infection develops (Moshe et al., 2016). This increase in virus amounts could be connected with the HSP90 activity in the cell protein degradation machinery. Indeed, HSP90 has a key role in the function of the 26S proteasome. Proteins destined for degradation are attached to ubiquitin (Ub) and this complex is then degraded by the 26S proteasome, the major proteolytic system of eukaryotic cells (Voges et al., 1999; Smalle and Vierstra, 2004; Sadanandom et al., 2012). In plants, loss of function of the 26S proteasome leads to cell death (CD) and to the release of ROS (Kim et al., 2003). GDA-induced HSP90 inhibition causes the disruption of the 26S proteasome and the loss of its protease activity (Nishizawa-Yokoi et al., 2010). HSP90 inactivation also leads to a decrease in the degradation of the TYLCV protein V2 by the 26S proteasome (Moshe et al., 2016). Loss of function of HSP70 and HSP90 had contrary effects on virus levels. As inhibition of *Hsp70* transcription by quercetin impaired the propagation of TYLCV, probably by slowing down the nuclear transport of the viral CP nuclear, GDA-treatment and *Hsp90* silencing inactivated the UPS, accompanied by increased viral CP and DNA levels.

### Involvement of Whitefly Chaperones in the Circulative Transmission of TYLCV

Whiteflies contain two types of chaperones: those synthesized by the insect cells and those produced by their endosymbiotic bacteria, housed in cells named bacteriocytes (Baumann, 2005). The cellular chaperones belong to the HSP family found in other eukaryotic cells (e.g., HSP23, HSP70, and HSP90). The expression of the *Hsp* genes is upregulated in response to abiotic (pesticide applications, heat, and UV radiation) and biotic stresses (viruses, bacteria, fungi, and insect natural enemies) (Zhao and Jones, 2012). The endosymbiotic GroEL chaperones are barrel-shaped structure consisting of two superimposed rings of seven subunits each. The co-chaperonin GroES is necessary for GroEL activity (Hayer-Hartl et al., 2016). GroEL and GroES are structurally and functionally nearly identical to the eukaryotic proteins HSP60 and HSP10, respectively.

The different whitefly *B. tabaci* species (De Barro et al., 2011) harbor the obligatory primary endosymbiont *Portiera aleyrodidarum*, together with some of the facultative secondary endosymbionts such as *Arsenophonus, Cardinium, Fritschea, Hamiltonella, Rickettsia*, and *Wolbachia* (Chiel et al., 2007). The role of an endosymbiotic GroEL protein in the circulative transmission of a plant virus by its insect vector was first demonstrated for aphid-transmitted luteoviruses (van den Heuvel et al., 1994). Later it was found that a GroEL produced by *B. tabaci* B (also known as MEAM1) *Hamiltonella* ensured the transmission of TYLCV to tomato plants by protecting the virus from destruction in the hemolymph (Morin et al., 1999). GroELs from other secondary endosymbionts, whether in B or Q (also referred as MED) whiteflies do not contribute to substantial level of TYLCV transmission (Gottlieb et al., 2010).

Other *B. tabaci* cellular chaperones may play a role in begomovirus transmission. A 16-kDa protein belonging to the HSP20 family interacted with TYLCSV CP in a protein-protein binding assay (Ohnesorge and Bejarano, 2009). Microarray studies indicated that the expression of an *Hsp70* gene was modulated upon TYLCV acquisition. TYLCV and HSP70 interacted in *in vitro* tests. TYLCV (and the bipartite begomovirus *Watermelon chlorotic stunt virus*, WmCSV) co-immuno-localized with HSP70 within epithelial cells of the whitefly midgut. Feeding whiteflies with an anti-HSP70 antibody was associated with an increase in the efficiency of virus transmission to plants, suggesting that HSP70 may help restrain virus translocation from the gut into the hemolymph (Götz et al., 2012). Interestingly HSP70 in *B. tabaci* behaves inversely than in tomato plants, where downregulation of *Hsp70* led to a decrease in TYLCV amounts (Gorovits et al., 2013a).

### Conversion of Cellular Chaperones from Soluble into Insoluble State upon TYLVCV Infection Is Part of the Cellular Protein Quality Control and Virus Degradation Process

**FIGURE 4:**
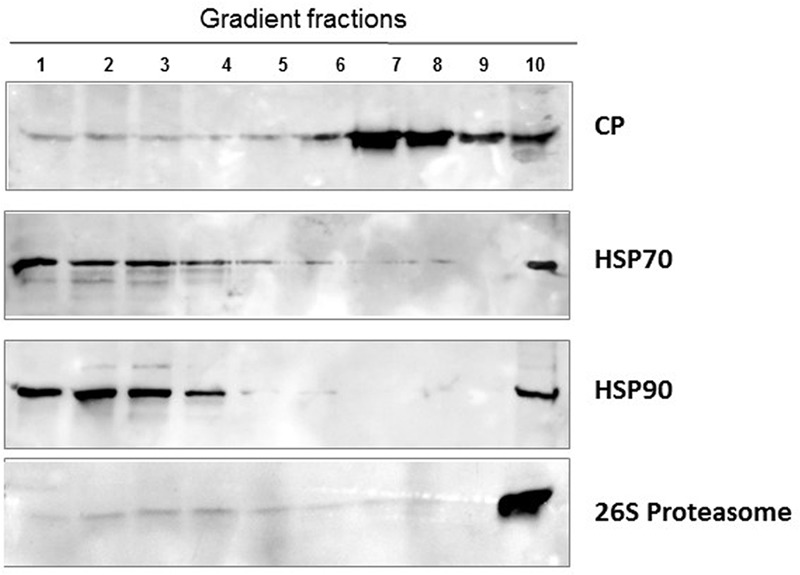
**Aggregates of TYLCV CP and whitefly HPS70 and HSP90, 26S proteasome.** Native proteins of viruliferous whiteflies (after about a week feeding on infected tomato) were separated by ultracentrifugation of 10–50% sucrose gradients. The gradients were resolved in 10 fractions. Aliquots were subjected to SDS-PAGE, followed by western blot immunodetection with antibodies against TYLCV CP, HSP70 and HSP90 and 26S proteasome. As in tomato, TYLCV induced the formation of large HSP70/90 aggregates; the 26S proteasome was also in large aggregates.

Western blot analysis of native proteins from non-infected and TYLCV-infected tomato leaves, separated by ultracentrifugation in 10–50% sucrose gradients, showed that HSP70 and HSP90 were found in aggregates only in infected tissues (**Figure 1**). The large aggregates also contained the other components of the plant PQC system, such as the autophagy-related protein 8 (ATG8) (Gorovits et al., 2016), ubiquitin, and the regulatory subunit of the 26S proteasome (Gorovits et al., 2014) (**Figure 1**). Since ATG8 is needed for the formation of autophagosomes and is a key element of autophagy (Shpilka et al., 2011), autophagy may be a major mechanism induced by plants to cope with TYLCV infection. Similarly, when viruliferous whiteflies native proteins were separated in sucrose gradients, the fractions containing large aggregates included also the 26S proteasome; ATG8 was not immuno-detected, perhaps because the antibody did not recognize the insect protein (**Figure 4**).

*In vitro* tests showed that large aggregates exhibited proteolytic activities that could digest all the six proteins encoded by the TYLCV genome. Moreover, incubation of detached tomato leaves with the 26S proteasome inhibitor MG132, with the autophagy inhibitor wortmannin and with the autophagy inducer rapamycin (Yang et al., 2013) caused changes in the TYLCV CP and V2 aggregation patterns, pointing on the involvement of these degradation mechanisms in TYLCV infection. The amounts of the six TYLCV proteins changed upon MG132 or wortmannin treatment, indicating that 26S proteasome and autophagy are involved in the degradation of begomoviruses (Gorovits et al., 2016). The presence of PQC elements, including crucial chaperones, indicated that these aggregates could be similar to animal VFs (Netherton and Wileman, 2011).

It was recently shown that TYLCV activated the autophagy pathway in the *B. tabaci* B (Wang et al., 2016). Upon feeding on infected tomatoes, there was a steady increase in the amounts of viral DNA and CP during the first 48 h followed by a decrease. Virus depletion was correlated with the activation of autophagy, as the levels of ATG8-II greatly increased and the amounts of autophagosomes in the whitefly midgut was enhanced. The activation of whitefly autophagy inhibited virus transmission, whereas the inhibition of autophagy promoted virus transmission. Hence, *B. tabaci* uses autophagy to curb TYLCV amounts in the insect, possibly to restrain putative negative effects of the virus on the whitefly life cycle (Wang et al., 2016).

## Discussion

### HSPs and PQC Elements Govern the Size of TYLCV-Containing Protein Aggregates, Which Serve to Protect or to Destroy TYLCV

Like the other begomoviruses, TYLCV is dependent on the host cell machinery for survival. During infection, begomoviruses remodel the host cells by interacting with cellular proteins. The limited set of viral proteins requires their multi-functionality and interaction with the host chaperones, such as HSP70 and HSP90. Protein aggregation is a major process in which viruses and viral proteins ensure their survival and replication in the infected cell. Based on their behavior in infected plants and viruliferous whiteflies, we suggest that movement, localization and degradation of the key chaperones (HSP70 and HSP90), together with PQC such as ubiquitin, 26S proteasome and autophagy proteins play a major role in TYLCV aggregation and consequently, in virus mobilization.

*Tomato yellow leaf curl virus* could induce a mechanism to sequester virus-induced misfolded or modified cellular proteins in aggregates to prevent the triggering of innate antiviral responses, inhibit the induction of cell death and prevent an activation of HSFs and their substrates, which suppress virus successful multiplication. Indeed, TYLCV-infected tomatoes are characterized by low levels of cell death; moreover, TYLCV is able to alleviate cell death, induced by the other stresses such as heat (Anfoka et al., 2016).

*Tomato yellow leaf curl virus* in tomato does not induce HSP70/HSP90 expression, but causes their shift from soluble proteins into aggregates (**Figure 1**). During the development of plant infection, HSP70 and HSP90 re-localize in an aggregated state, from the cytoplasm to the nucleus in the cells associated with the vascular system. The other PQC elements such as ubiquitin, 26S proteasome subunits, autophagy protein ATG8 are present in the large nuclear VFs together with TYLCV proteins (mainly CP), viral DNA, DNA-protein complexes and infectious virions.

Intracellular homeostasis depends on PQC, the constant degradation and re-synthesis of proteins. At one end, chaperons modulate protein folding and repair. At the other end, HSPs are involved in proteasome and autophagy removal of dysfunctional proteins. These systems may influence each other (Dokladny et al., 2013, 2015). Invading viruses are considered by cells under attack as entities to be destroyed or sequestered, while viruses have evolved mechanisms to subvert proteolysis, such as the autophagic response (reviewed by Chiramel et al., 2013). Apart from playing a major role in antiviral defense, autophagy can also enhance viral replication (Shoji-Kawata and Levine, 2009). Inhibitory effect of autophagy genes was demonstrated on *Tobacco mosaic virus* (TMV) replication in *N. benthamiana* plants (Liu et al., 2005). In *B. tabaci* B, TYLCV triggers the insect autophagy antiviral program, promoting the formation of autophagosomes and curbing TYLCV infection (Wang et al., 2016).

HSP70 co-localized with TYLCV CP in aggregates not only in tomato plants, but also in the TYLCV *B. tabaci* vector. Whiteflies are well adapted to high temperatures. When temperatures rise from 25 to 40°C, the expression of their *Hsp70* and *Hsp90* genes is upregulated, improving the fitness of the insect (Mahadav et al., 2009; Díaz et al., 2015). In contrast, the presence of TYLCV impairs the fitness of *B. tabaci* raised at high temperatures.

The completion of a successful infection by the begomovirus TYLCV depends on its interaction with cellular chaperones, among them HSP70 and HSP90, at all stages of the virus life cycle: in the tomato host, during acquisition by its whitefly vector and in the insect. The outcome of which was the formation of protein aggregates of different sizes, which could simultaneously serve to protect and destroy TYLCV, depending on the recruitment of PQC components.

### Do Changes in the Amounts of Plant HSPs, and Thereby in the Size of TYLCV-Containing Aggregates, Modulate the Acquisition of TYLCV by *B. tabaci*?

**FIGURE 5:**
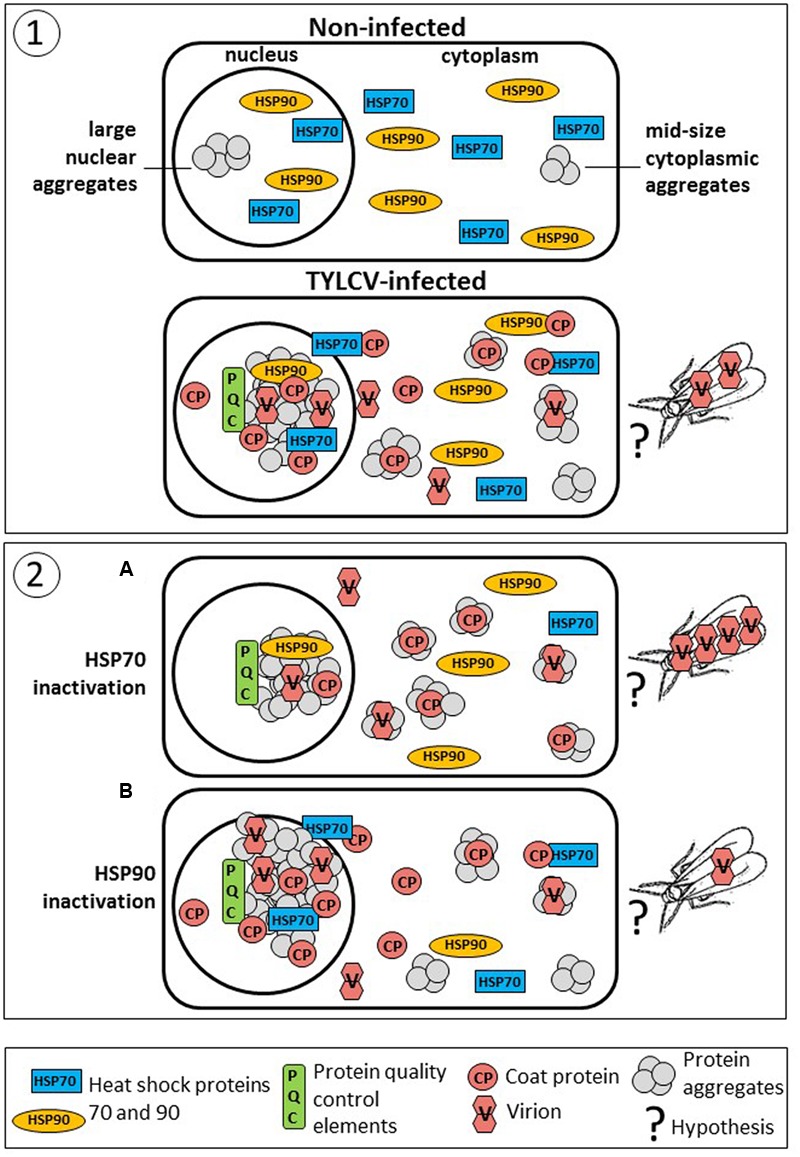
**Diagram summarizing the association of TYLCV with tomato host chaperons and how it may influence efficacy of virus acquisition by the whitefly vector.** Panel (1): TYLCV causes changes in the amounts of plant chaperons and their intra-cellular location. Non-infected cells contain pool of free nuclear and cytoplasmic HSP70 and HSP90. Upon TYLCV infection there is a decline in chaperons amounts (Gorovits et al., 2007; Moshe et al., 2012). At the same time, infection leads to massive protein aggregation in the nucleus and cytoplasm. Large nuclear aggregates contain viral components (mainly, CP) together with virions (Gorovits et al., 2013b), and cellular proteins, including HSP70 (Gorovits et al., 2013b) and HSP90 (Moshe et al., 2016), as well as the other PQC elements, as in viral factories in mammalian cells. Mid-size cytoplasmic aggregates contain CP, but not HSP70 and HSP90. Whiteflies may acquire free virions or virions detached from aggregates that moved to the phloem. In the insect, TYLCV CP is localized with HSP70 in aggregates present in the filter chamber (Götz et al., 2012). Panel (2): The amounts and activities of chaperons influence the amounts of viral particles and their degree of aggregation in such a way that they may modulate TYLCV acquisition by whiteflies. (A) Inactivation of HSP70 leads to a decline in virus amounts, especially in nucleus, but results in increased abundance of CP and DNA (virions) in mid-size cytoplasmic aggregates (Gorovits et al., 2013a). Hence, it is likely that there are more virions in the phloem, and acquisition of increased virus amounts by whiteflies. (B) Inactivation of HSP90 leads to a significant increase in the total virus amounts especially in nuclear aggregates, but to decrease of virus in cytoplasmic aggregates (Moshe et al., 2016). Therefore, we hypothesize that since most virus is trapped in nuclear aggregates, less virus may reach the phloem, and therefore less virus is acquired by whiteflies.

Inhibitors of HSP70 and HSP90 are able to change the size and the pattern of protein aggregation in infected tomato, together with their content in viral DNA and CP (**Figure 5**). In tomato leaves with quercetin-inactivated HSP70, CP shifted from large to mid-size aggregates, accompanied by a significant decrease of the viral DNA amounts present in untreated plants (Gorovits et al., 2013a). On the contrary, in leaves with GDA-inactivated HSP90, CP shifted from mid-size to large aggregates that contained higher amounts of viral CP and DNA (Moshe et al., 2016). Large aggregates are confined to the nucleus, while midsized aggregates are present mostly in the cytoplasm. Whiteflies acquire virus from the phloem of infected plants. Therefore, virions need to move from the phloem-associate cells to the plant vascular system. Although it was shown that nuclear large aggregates contain infectious particles (Gorovits et al., 2013b), it is doubtful that large aggregates-containing virions translocate as such to the phloem. Therefore it is likely that the viral particles acquired by whiteflies originate from cytoplasmic free virus or/and from virus bound to mid-size aggregates. It is possible that virions move in an out from the nucleus. In, to find a shelter to avoid destruction and to serve as template for replication, and out, to provide free virions and virions attached to mid-size aggregates that will serve as inoculate in whitefly-mediated transmission. If this is the case, it might be possible to impair the ability of whiteflies to acquire begomoviruses by increasing the relative amounts of large aggregates with HSP90 inhibitors and by applied various abiotic stresses causing the formation of these TYLCV-containing structures (**Figure 5**).

## Author Contributions

All authors listed, have made substantial, direct and intellectual contribution to the work, and approved it for publication.

## Conflict of Interest Statement

The authors declare that the research was conducted in the absence of any commercial or financial relationships that could be construed as a potential conflict of interest.
